# Outcome in methadone maintenance treatment of immigrants from the former Union of Soviet Socialist Republics

**DOI:** 10.1186/s12954-024-00970-7

**Published:** 2024-03-01

**Authors:** Ayali Noya, Sason Anat, Schreiber Shaul, Adelson Miriam, Peles Einat

**Affiliations:** 1 Dr. Miriam and Sheldon G. Adelson Clinic for Drug Abuse, Treatment and Research, Tel-Aviv Sourasky Medical Center, Tel Aviv, Israel; 2 Faculty of Medical and Health Sciences, Tel Aviv University, Tel Aviv, Israel; 3 Sagol School of Neuroscience, Tel Aviv University, Tel Aviv, Israel

**Keywords:** Methadone maintenance, Immigrants, Former USSR, Retention, Survival

## Abstract

**Context:**

Immigrants from the former Union of Soviet Socialist Republics (USSR) are more prevalent in Methadone maintenance treatment (MMT) in Israel than their percentage in the general population.

**Aims:**

To compare their characteristics and outcomes to those of Israeli-born and other immigrant patients.

**Methods:**

Retention and survival since admission (June/1993–Dec/2022) until leaving treatment (for retention), or at the end of follow-up were analyzed. Vital data was taken from a national registry. Predictors were estimated using Kaplan–Meier and Cox regression models.

**Results:**

The USSR patients (N = 262) compared with other immigrants (N = 132) and Israeli-born (N = 696) were more educated (≥ 12y) (*p* < 0.001), admitted to MMT at a younger age (*p* < 0.001), following a shorter duration of opioid usage (*p* < 0.001). More of them ever injected drugs (*p* < 0.001) and ever drank alcohol (*p* < 0.001). One-year retention was comparable (77.2% vs. 75.6% and 72%, *p* = 0.2) as did opioid discontinuation in those who stayed (*p* = 0.2). Former USSR patients had longer cumulative retention of their first admission (*p* = 0.05) with comparable overall retention since first admission, and survival, although the age of death was younger. Specific origin within the former USSR found immigrants from the Russian Federation with the best outcome, and those from Ukraine as having high HIV seropositive and shorter retention.

**Conclusions:**

Despite several characteristics known to be associated with poor outcomes, former USSR immigrants showed better adherence to MMT, reflected by their longer cumulative retention in their first admission, lower rate of readmissions, and a comparable survival and overall retention in treatment. An in depth study is needed in order to understand why they decease at a younger age.

## Introduction

Methadone maintenance treatment (MMT) is considered the most safe and effective treatment for opioid-use disorders [1, 2]. It is strongly associated with reducing illicit opiate use, decreasing criminal behaviors, improving mental and physical well-being, decreasing morality as well as reducing other complications [2]. MMT requires long-term or even lifelong daily intake of methadone. Therefore, longer retention in treatment is a key factor in achieving better outcomes and is considered the best predictor for success in MMT evaluation [3]. Previous studies from all over the world that examined retention rates in MMT show that retention is influenced by various factors that can be divided into three main elements: individual, program (methadone dosage, treatment accessibility), and social (society, family, peers' support) [4].

The individual-related factors are mostly sociodemographic and include age, race, religious faith, opiate use history, criminal history, mental health status, education level, and marital status. It has been shown that longer MMT retention is predicted by older age (> 30 years), being married, or living with a stable partner, being highly educated (> 8 years), and having a job. Ethnicity and gender factors are inconsistent in predicting MMT retention [3, 5–12].

Israel is a Jewish state, where non-Jews and Jews live together. Specifically, 73.6% of the total population are Jews, 21.1% are Arabs (of them 83.4% are Muslims, 8.4% Christians, 8.2% Druze) and 5.3% are non-Arab Christians and persons of other religions [13]. The state of Israel is an immigrant-receiving country for Jews from 170 countries and nations worldwide [14].

In Israel, immigrants from the former Soviet Union are notably more prevalent among the substance use disorder population than their percentage in the general population [15]. Following the disintegration of the USSR (1991), more than a million people (Jews and non-Jews but of Jewish origin) immigrated to Israel becoming the largest of the immigration groups in Israel, ever. The immigrants arrived from the various states and republics that gained independence (including the Russian Federation itself), they all spoke the Russian language, but differed in many aspects (i.e., native language, tradition, and culture, etc.), characteristics of the country of origin (traits maintained despite of the 1922 annexation to-, or occupation by- the USSR) [16, 17]. At that time, the MMT was available in Israel for many years, as in other Western countries, but not in the USSR, whose HIV prevalence was very high. Nowadays, because of the Ukraine-Russian war, a new wave of immigrants mainly from Ukraine arrived in several countries, including Israel. However, while buprenorphine maintenance treatment was introduced in Ukraine in 2004 and MMT in 2008 [18], it is still banned in Russia [19]). Based on the high OUD and high HIV prevalence (around 25%) in Ukraine [20] our data from the previous immigrant wave, may be relevant to the current wave as well, although their proportion as OUD in the Israeli population is still not clear, as an increase in opioid usage over the last years was observed in Israel population, reflected by the fentanyl opioid prescription rise [21].

Our aim in this study is to compare the characteristics and success in MMT of opioid use disorder individuals from the former USSR, divided into three major groups: the Russian Federation (including the Caucasus parts of the Federation), Ukraine, and the rest, to Israeli-born individuals and immigrants from other countries.

## Methods

All patients signed an inform consent on admission to MMT and the study analyses was approved by the local Medical Center Helsinki committee (IRB).

### Study population

The methadone maintenance treatment (MMT) clinic was established in 1993 and treats Individuals > 18 years of age diagnosed with DSM-IV or DSM-5 opioid use disorder who have experienced at least two institutional detoxification failures. All patients ever admitted to the clinic (were administered at least one daily methadone dose) since 1993 and until December 2022 (N = 1098) except 8 patients whose immigrant status was unknown, were studied; a total of 1090 patients. Patients’ retention since first admission, overall retentions, and survival since first admission and until August 2022 were calculated.

The vital status of each patient who left treatment was checked individually on the Israel National Population Registry which records all deaths in the country, excluding 18 who were missing. The 1072 patients had a total of 14,013.2 person-years (py) (9251.6 Israeli, 1061.1 Russian Federation, 14,119.1 Ukraine, 611.1 other former USSR, and 1677.5 Other immigrants) of follow-up (mean 13.1 ± 8.5 years and a total of 5461.1 py under observation (in MMT) (mean 11.3 ± 8.2 years).

### The MMT program

Patients arrived either on their account or following a referral from the affiliated Tel Aviv Sourasky Medical Center wards or Emergency, or other sources, and voluntarily come to the clinic every day to receive their daily dose of methadone. After a minimum of 3 months of drug abstinence and based on behavior, patients may "earn" the right to achieve "take-home" methadone doses, up to a maximum of two weeks following 2 years of uninterrupted abstinence. As part of the treatment at the clinic, each patient receives regular personal counseling and psychosocial therapy, medical follow-up, and group therapy meetings (described in detail [22]).

### Study variables

Demographic and sociodemographic data were collected from patients' charts.

### Urine toxicology and methadone

Twice a month random urine samples were routinely collected. Each subject's urine tests were analyzed for opiates, cocaine metabolite (benzoylecgonine), benzodiazepines (BDZ), amphetamines, and cannabis. "Positive" classification for each drug is defined on admission to MMT or if at least one urine sample for any drug was positive during the first month or after 1 year of treatment. Methadone dosage on the 13th month was used for analyses, or at the last month of treatment among patients who stayed at least 3 months but left before one year in MMT.

### Cause of death

The cause of death was taken from each deceased patient's chart or from a national population registry, whichever was more detailed.

### Statistical analyses

Data analyses were performed using SPSS version 29 (SPSS Inc. Chicago, Illinois). Comparison between groups was done with chi-square for categorical variables and ANOVA for continuous variables. Logistic regression comparing USSR and Israeli-born with all significant variates (*p* < 0.05) in univariate analyses was calculated. Survival and retention were calculated from the first MMT admission until the patient died or left, respectively, or until the end of follow-up using Kaplan Meier survival analyses (results given as mean and 95% confidence interval [95%CI]). The Log Rank test was used to test for the statistical significance for the results of the examined variables. The variables that were found to significantly predict survival or retention in a Kaplan–Meier analysis (*p* < 0.05) were included in the multivariate model (Cox) for survival and retention.

## Results

### *Characteristic differences *(Table 1)

**Table 1:** Characteristics of former USSR, Israeli-born, and other immigrant patient groups

	Former USSR N = 262			Israeli born	Other immigrants	*p* (F)**
	Russia Federation	Ukraine	Others USSR			
N (%)	89 (100)	110 (100)	63 (100)	696 (100)	132 (100)	
Female*	31 (34.8)	30 (27.3)	16 (25.4)	159 (22.8)	18 (13.6)	0.002 (13.0)
Jew faith*	58 (66.7)	73 (66.4)	49 (81.7)	634 (91.2)	106 (80.3)	< 0.001 (64.4)
Age at admission, y	35.5 ± 8.7	35.5 ± 9.1	38.2 ± 10.7	42.4 ± 10.2	44.7 ± 11.5	< 0.001 (49.1)
Age < 30 y on admission	21 (23.6)	27 (24.5)	16 (25.4)	64 (9.2)	14 (10.5)	< 0.001 (36.3)
In Israel pre- MMT, y	13.4 ± 10.6	11.1 ± 9.0	16.6 ± 10.7		31.3 ± 19.3	< 0.001 (144.5)
pre- MMT > 10y*	38 (43.7)	59 (55.1)	20 (33.3)		26 (22.4)	< 0.001
Immigration year						< 0.001)215)
≤ 1989	10 (11.2)	6 (5.5)	7 (11.1)		85 (64.4)	
1990–1999	62 (69.7)	77 (70.0)	43 (68.3)		24 (18.2)	
2000–2003	11 (12.4)	21 (19.1)	5 (7.9)		2 (1.5)	
2004–2013	3 (3.4)	3 (2.7)	5 (7.9)		5 (3.8)	
2014–2022	2 (2.2)	1 (0.9)	1 (1.6)		5 (3.8)	
unknown	1 (1.1)	2 (1.8)	2 (3.2)		11 (8.3)	
Age at opioid onset, y	21.3 ± 6.2	22.3 ± 7.4	20.9 ± 6.3	22.3 ± 7.5	25.1 ± 8.5	< 0.001 (11.8)
Age at alcohol onset, y	14.9 ± 2.1	17.3 ± 5.3	15.2 ± 3.3	18.5 ± 7.0	17.3 ± 7.9	0.001 (6.7)
Ever alcohol use*	50 (83.3)	60 (78.9)	32 (69.6)	225 (57.8)	47 (64.4)	< 0.001 (22.7)
Age at cannabis onset, y	18.4 ± 6.8	18.2 ± 6.0	17.0 ± 5.5	16.8 ± 6.0	16.5 ± 5.5	0.1
Ever cannabis usage*	79 (88.8)	90 (81.8)	55 (87.3)	566 (81.7)	110 (82.7)	0.5
Age at BDZ onset, y	25.6 ± 8.1	27.1 ± 7.7	25.4 ± 8.0	27.5 ± 10.6	31.1 ± 13.3	< 0.001 (7.5)
Ever BDZ usage*	72 (80.9)	85 (78.0)	45 (71.4)	561 (81.7)	109 (82.0)	0.2
Pre-MMT opiate, y	14.2 ± 9.0	13.1 ± 8.0	17.1 ± 10.6	20.3 ± 10.5	19.5 ± 12.4	< 0.001 (29.0)
Opiate abuse ≥ 20 y*	23 (25.8)	25 (22.9)	24 (40.7)	351 (51.7)	61 (48.8)	< 0.001 (48.1)
Way of admission*						< 0.001 (30.1)
Self	61 (68.5)	52 (47.7)	34 (54.8)	469 (67.9)	78 (60.5)	
Medical/hospital	(18.0) 16	31 (28.4)	17 (27.4)	84 (12.2)	15 (11.6)	
Others	12 (13.5)	26 (23.9)	11 (17.7)	138 (20.0)	36 (27.9)	
DSM-5 Psychiatric*						0.002 (19.6)
Axis I	15 (18.3)	17 (17.0)	13 (23.2)	98 (15.3)	15 (12.4)	
Axis II	25 (30.5)	42 (42.0)	16 (28.6)	286 (44.5)	59 (48.8)	
Axis I & II	22 (26.8)	19 (19.0)	8 (14.3)	159 (24.8)	31 (25.6)	
None	20 (24.4)	22 (22.0)	19 (33.9)	99 (15.4)	16 (13.2)	
Living alone*	63 (70.8)	80 (72.2)	43 (68.3)	466 (67.2)	88 (66.2)	0.8
Having children ≥ 1*	41 (46.1)	62 (56.4)	34 (54.0)	478 (69.0)	85 (63.9)	< 0.001 (23.9)
Education ≥ 12y*	42 (47.2)	49 (45.4)	26 (43.3)	142 (20.9)	39 (29.8)	< 0.001 (51.1)
Criminal history events	27.8 ± 42.1	22.9 ± 39.6	15.6 ± 32.9	31.8 ± 43.1	39.0 ± 49.1	0.007 (5.0)
Ever injected*	72 (80.9)	100 (90.9)	52 (83.9)	343 (49.9)	64 (48.5)	< 0.001 (121.1)
Hepatitis C antibody*	69 (80.2)	83 (77.6)	46 (75.4)	303 (45.6)	57 (44.5)	< 0.001 (90.0)
HIV antibody^≠≠^	10 (11.2)	35 (31.8)	8 (12.7)	18 (2.6)	6 (4.5)	< 0.001 (90.0)
Hepatitis B antigen^≠≠^*	5 (5.6)	9 (8.2)	4 (6.3)	12 (1.7)	6 (4.5)	0.01 (14.8)
***Drug on admission***						
Benzodiazepine*	55 (61.8)	59 (53.6)	33 (52.4)	409 (58.9)	75 (56.8)	0.8
Cocaine*	19 (21.3)	32 (29.1)	20 (31.7)	209 (30.1)	37 (28.0)	0.5
THC*	16 (18.0)	15 (13.6)	9 (14.3)	94 (13.5)	15 (11.4)	0.7
Amphetamines^#^*	4 (4.7)	4 (3.8)	4 (7.0)	42 (6.3)	9 (7.0)	0.8
***After one year***						
Retention* (*%*)	83.1	76.1	78	72	75.6	0.2
Opioid stopped* (%)	71.6	74.7	71.7	66.3	70	0.2
Benzodiazepine* (%)	45.9	49.4	37	49.5	45	0.7
Cocaine* (%)	23	18.1	15.2	18.3	18.5	0.4
THC* (%)	29.7	18.1	15.2	15.1	11	0.002 (12.3)
Methadone dose (mg/d)	126.2 ± 44.7	129.2 ± 48.0	130.6 ± 44.9	121.8 ± 44.3	119.3 ± 42.4	0.08 (2.5)

^*^Chi square **Comparison between former USSR, Israeli born and other Immigrants. ≠ 115 were missing ≠  ≠ 44 were missing #54 were missing

The immigrants from the former USSR included 89 from the Russian Federation (among them 7 from the Caucasus parts of the Federation), 110 from Ukraine, and 63 from several other former USSR Republics (6 Azerbaijan, 4 Belarus, 18 Georgia, 10 Kazakhstan, 1 Latvia, 2 Lithuania, 6 Moldova, 2 Turkmenistan, 14 Uzbekistan) that compared with other immigrants (N = 132) (76 from Africa/Asia, 37 from Europe/America, 19 from the Palestinian authority) and Israeli-born (N = 696) are presented in Table 1.

### *Characteristic differences between former USSR, other immigrants, and Israeli-born subgroups *(Table 1)

The 262 immigrants from the former USSR (the Russian Federation n = 89, Ukraine, n = 110, Others n = 63) compared with immigrants from other countries (N = 132) and Israeli-born (N = 696) had more females (*p* = 0.002), fewer were of Jewish faith (*p* < 0.001), and they were more educated (*p* < 0.001). They were admitted to treatment at a younger age (*p* < 0.001), following a shorter duration of opioid usage (*p* < 0.001), with fewer criminal history events (*p* = 0.007). More of them ever injected drugs (*p* < 0.001), ever drank alcohol (*p* < 0.001) that started at a younger age (*p* < 0.001). More of them had no psychiatric comorbidity (*p* = 0.002), more of them were referred to MMT from a medical facility, and fewer arrived following self-referral (*p* < 0.001). Fewer had children (*p* < 0.001) and more were hepatitis C and HIV sera positive (*p* < 0.001 for each) and had hepatitis B antigen (*p* = 0.01). Drugs in urine did not differ on admission (BDZ, cocaine, cannabis, amphetamines). Age of opioid onset, and BDZ onset, differed between the groups (*p* < 0.001 for each), and were both younger among former USSR immigrants than the immigrants from other countries, with no differences from the Israeli-born group (0.2 for each). Most of the former USSR immigrated to Israel between 1990–1999 compared to others who immigrated mostly before 1990 (*p* < 0.001). The former USSR patients admitted to MMT following a shorter duration since immigration than the immigrants from other countries (*p* < 0.001).

### *Logistic regression multivariate analyses *(Table 2)

**Table 2:** The USSR vs. Israeli born group (Logistic regression model)

	*p* value	Exp(B)	95% CI for Exp(B)	
			Lower	Upper
Axis I only	.005	2.588	1.343	4.988
No Axis I & II	< .001	5.473	2.562	11.694
Non-Jew faith	< .001	3.314	1.706	6.441
Not having children	.016	1.928	1.130	3.288
Educated ≥ 12 years	< .001	3.927	2.294	6.723
Opioid usage < 20y	< .001	3.035	1.708	5.390
Alcohol history	.004	2.315	1.313	4.080
Age admitted < 30y	.009	2.964	1.307	6.718
Ever drug injected	< .001	2.979	1.557	5.701
Hepatitis C antibody	< .001	3.900	2.045	7.438
HIV antibody	.004	4.153	1.556	11.082
Constant	< .001	.004		

Being from the former USSR and not Israeli-born was 2.6 more likely if patients had any DSM-5 Axis I psychiatric diagnosis, 5.5 more likely with no DSM-5 Axis I&II psychiatric diagnosis, 3.3 more likely if they were non-Jews, 1.9 more likely if they had no children, 3.9 more likely if they had ≥ 12 years of education, 3 times more likely if admitted younger than 30y and 3 times more likely following < 20 years of opioid usage, 2.3 more likely if they ever drank alcohol, and 3.9 more likely having hepatitis C and 4.1 more likely having HIV antibodies (Table 2).

### *Characteristic differences between former USSR subgroups *(Table 1)

The immigrants from the former USSR (The Russian Federation n = 89, Ukraine, n = 110, Others n = 63) (Table 1) did not differ by females’ proportion (*p* = 0.4) and age of admission to treatment (*p* = 0.1), nor by years of education (*p* = 0.9), neither being alone (0.9), having children (0.3), way of admission (*p* = 0.06), psychiatric comorbidity (*p* = 0.2) or substance proportion on admission (p > 0.4). The Ukraine group was admitted to MMT following the shortest duration since immigration (p(F = 5.8) = 0.003). The proportion of Jewish faith was higher (p(Chi square 4.7) = 0.03) in those from other former USSR territories than in those from the Russian Federation or the Ukraine immigrants. They were also admitted to treatment following a longer duration of opioid usage (p(F = 4.5) = 0.02) as compared with the Russian and Ukrainian immigrants. The proportion of alcohol drinking history was comparable between the groups (*p* = 0.2), but the age of drinking onset was older among the Ukraine than the two other groups (p(F = 5.8) = 0.004). However, more of the Ukraine immigrants were HIV antibody positive (p(Chi square 4.1) = 0.001), with a trend of higher ever drug injection (*p* = 0.1), but with a comparable proportion of hepatitis C antibody positive (*p* = 0.8) and hepatitis B antigen (*p* = 0.7).

### *Treatment outcomes, one year *(Table 1)

#### Comparison between former USSR, other immigrants, and Israel-born subgroups

Retention rate after one year did not differ significantly between the former USSR immigrants’ group and Israeli-born or other immigrants’ group (77.2%, 72%, and 75.6% respectively, *p* = 0.2), neither differed methadone dose (128.5 ± 46.0, 121.8 ± 44.3, 119.3 ± 42.4, *p* = 0.08), and groups also did not differ in the proportion of opioids discontinuation in those who stayed at least 1 year (73%, 66.3%, and 70% respectively *p* = 0.2). The proportions of being positive to BDZ (46.1%, 49.5%, and 45%, *p* = 0.7) cocaine (22.3%, 18.3%, and 18.5%, *p* = 0.4) after one year did not differ between groups, but cannabis use was more prevalent among those from the USSR (22.5%) than the other immigrants (15.1%) and the Israeli-born (11%, *p* = 0.002). Retention rate did not differ by short (< 10 years) or long duration (≥ 10 years) in Israel before admission to MMT by any group, except in those with a short duration < 10y, a lower retention rate was found among the immigrants from other countries group (56%) than the former USSR (80.3%, *p* = 0.02).

#### *Comparison between former USSR subgroups *(Table 1)

Within former USSR subgroups retention after 1 year did not differ (Russian Federation *p* = 0.5) as opioid discontinuation (*p* = 0.9). The proportions of being positive to BDZ (*p* = 0.4) cocaine (*p* = 0.5), and cannabis (*p* = 0.1) after one year did not differ between groups, and their methadone dose (*p* = 0.9) (Table 1).

### Cumulative retention

**Fig. 1:**
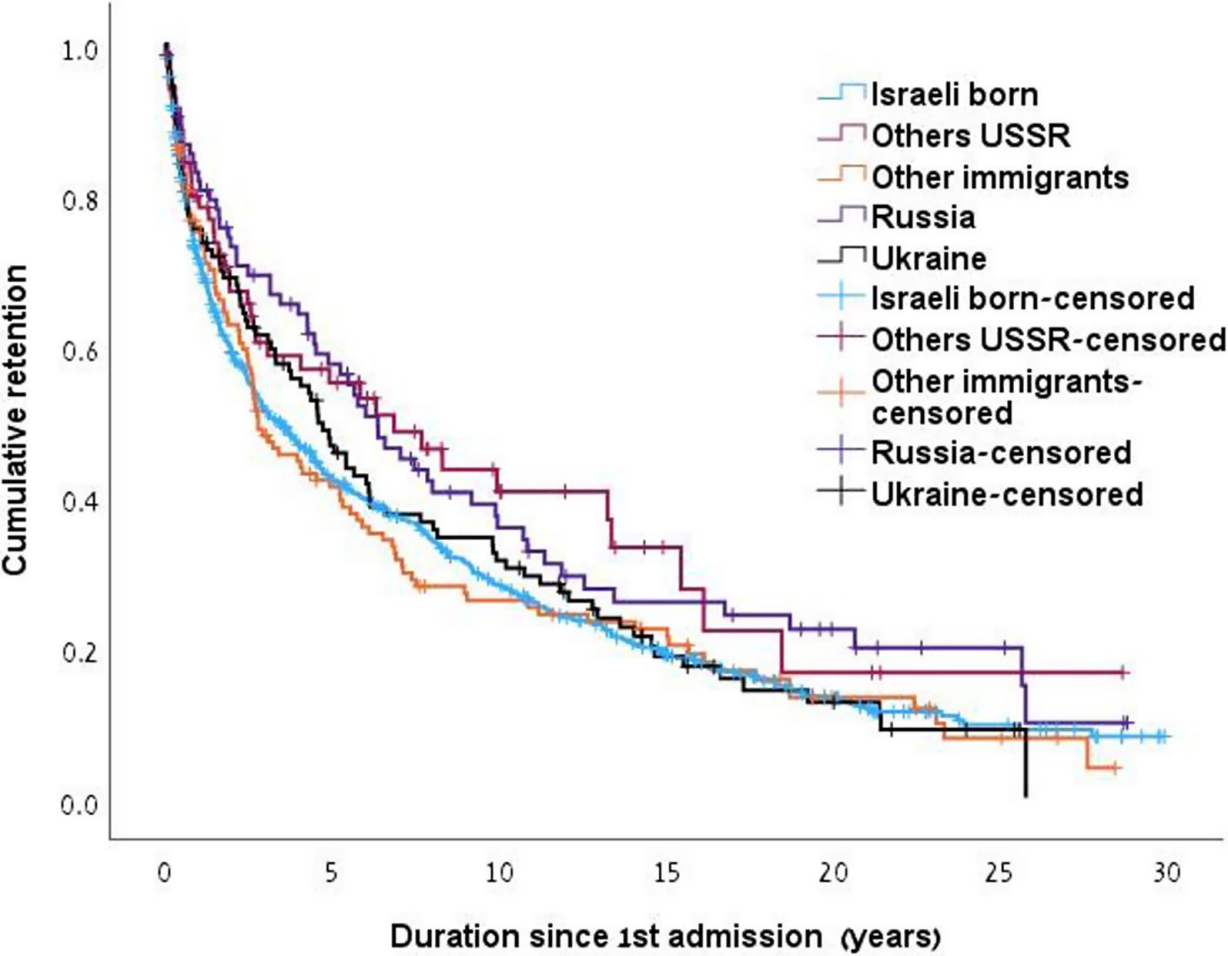
Cumulative retention in MMT since first admission by study groups. (Israeli born 7.7y (95% CI 7–8.4) vs. Others USSR 10.3 y (7.3–13.3, p = 0.04). Other immigrants 7.4y (95% CI 5.8–9.0). Russia 10.1 (7.8–12.4) vs. Israeli born (*p* = 0.03), and vs. other immigrants (*p* = 0.04). Ukraine 7.8y (95% CI 7.8–9.4))

**Fig. 2:**
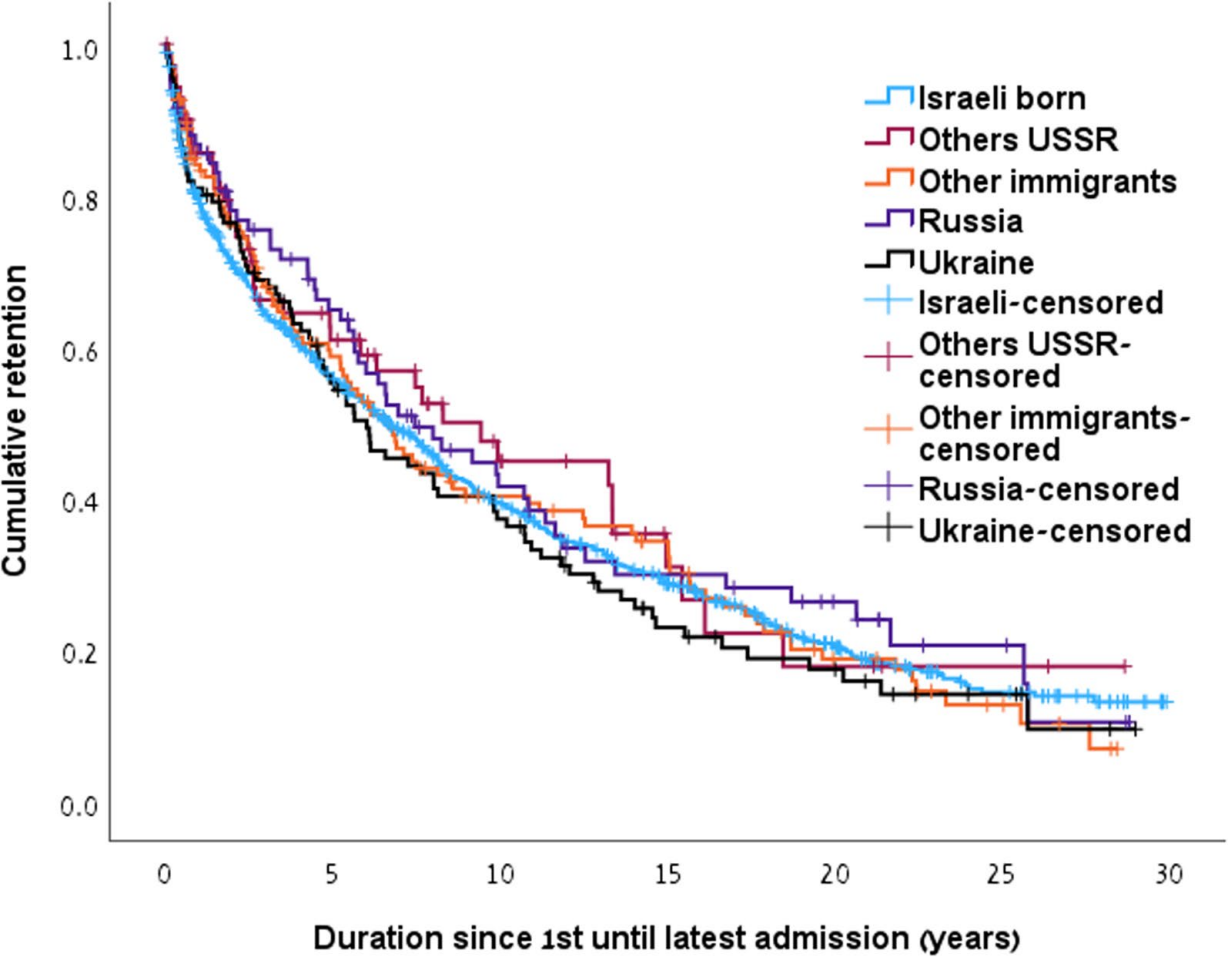
Cumulative retention in MMT since admission until latest admission by study groups. (Israeli born 10.3y (95% CI 9.5–11.1), Others USSR 11.2y (95% CI 8.3–14.0), Other immigrants 10.2y (95% CI 8.5–11.9), Russia 11.1y (8.8–13.4), Ukraine 9.5y (95% CI 7.6–11.3))

Former USSR immigrants had significantly longer cumulative retention 9.0y (95% CI 7.6–10.2) than the Israeli-born group 7.7y (95%CI 6.9–8.4) p(chi square 4.7) = 0.03) and a trend of higher with the other immigrants (7.3y (95% CI 5.7–8.9, *p* = 0.09). Comparing the 5 groups (Fig. 1), Of the former USSR groups, the Russian Federation had significantly longer cumulative retention than the Israeli-born (*p* = 0.03) and then the other immigrants. The other USSR group also had longer cumulative retention than the Israeli-born group (*p* = 0.04). The readmissions were less prevalent among the former USSR immigrants (11.7%) than that among the other immigrants 27.8% and the Israeli-born individuals 21.9%, *p* < 0.001). Within former USSR groups, readmission was lower among each of the 3 groups (Russian Federation, 10.1%, Ukraine, 11.8% and the other USSR, 15.9%, *p* = 0.6). Thus, a comparison in cumulative retention since 1st admission and until left at the latest admission or the end of follow-up was done, showing comparable cumulative retention between the groups; USSR, Israeli-born, and other immigrants had cumulative retention of 10.0y (95%CI 8.7–11.2), 9.4y (95%CI 8.7–10.1) and 9.6y (95%CI 7.9–11.2, *p* = 0.07) respectively. The 5 groups also did not differ (Fig. 2). When dividing the 30 years cohort into patients who were admitted in the early- 1993–1999 (n = 273), middle- 2000–2009 (n = 442), and late- 2010–2022 (n = 381) periods, the former USSR immigrants had significantly superior cumulative retention than the other groups in the late period (*p* = 0.03) and a comparable one in the early (*p* = 0.2) and middle (*p* = 0.9) periods, in the first admission, and comparable cumulative retention since the 1st admission and until leaving treatment at the latest admission, or at the end of follow-up, in all the 3 periods (Table 3).

**Table 3:** Cumulative retention (1st and overall admissions) by admission period groups and by former USSR, Israeli-born, and other immigrant patient groups

Periods	1st admission	*p* value	Overall admissions	*p* value
	Mean (95% CI)		Mean(95% CI)	
*Early *(*1993–1999*)				
Israel	7.8 (6.5–9.1)	0.2	11.8 (10.4–13.3)	0.3
Former USSR	10.6 (7.3–14.0)		13.9 (10.5–17.4)	
Other Immigrants	7.1 (4.3–9.8)		10.6 (7.7–13.6)	
*Middle *(*2000–2009*)		0.9		0.8
Israel	8.3 (7.3–9.3)		10.1 (9.0–11.1)	
Former USSR	8.4 (7.0–9.8)		9.5 (8.1–11.0)	
Other Immigrants	8.2 (6.1–10.4)		10.5 (8.3–12.6)	
*Late *(*2010–2022*)		0.026		0.4
Israel	4.3 (3.6–4.9)		5.6 (4.8–6.3)	
Former USSR	5.8 (4.8–6.9)		6.2 (5.2–7.2)	
Other Immigrants	4.1 (2.6–5.6)		5.7 (4.0–7.3)	

### Survival

**Fig. 3:**
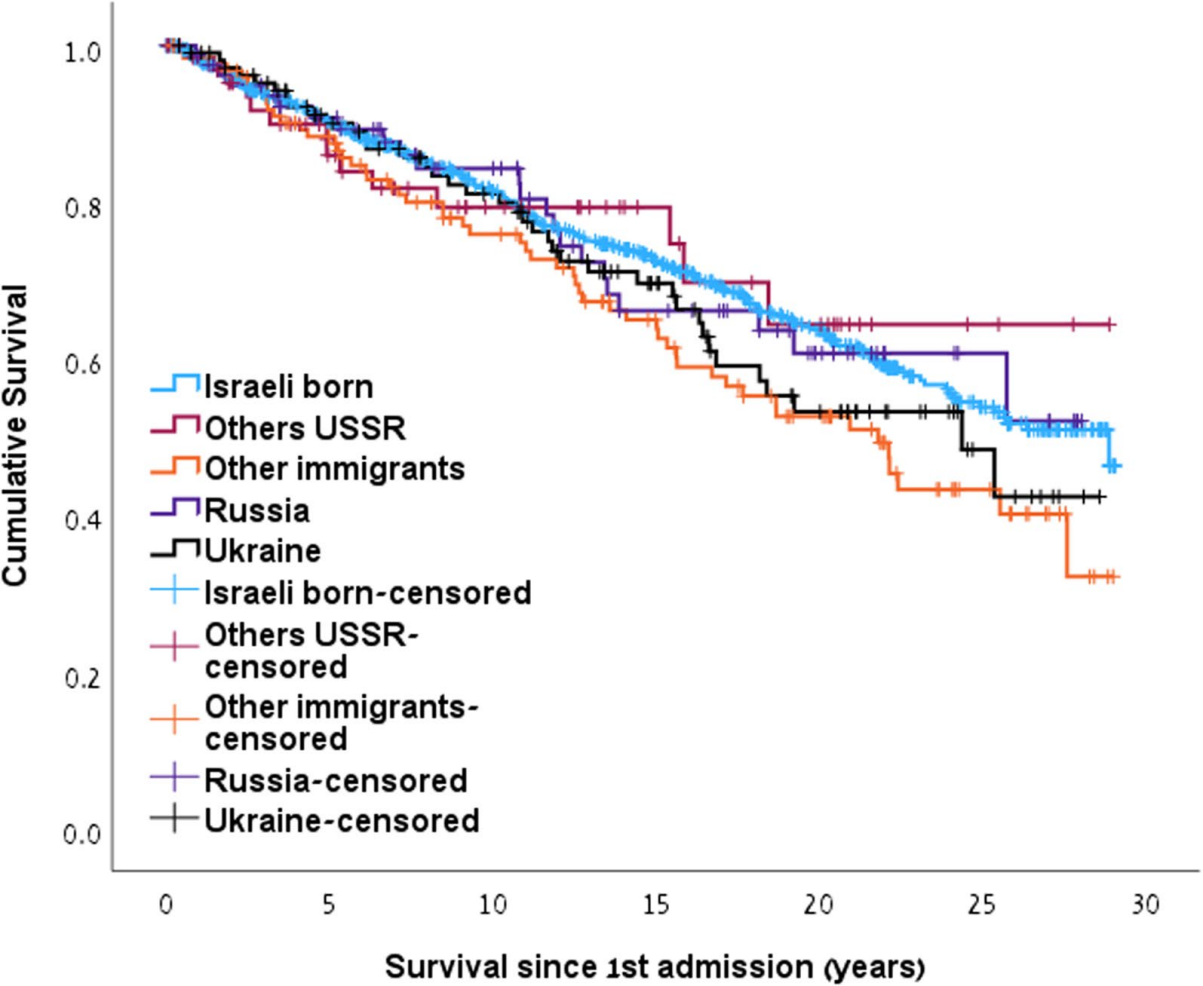
Cumulative survival since admission to treatment by study groups. (Israeli born 21.3y (95% CI 20.4–22.1), Others USSR 22y (95% CI 18.8–25.0), Other immigrants 18.9y (95% CI 17–20.8) vs. Israeli born (*p* = 0.03), Russia 20.7y (95% CI 18.3–23.1), Ukraine 19.9y (17.9–22.0))

The survival since admission to MMT and until death or end of follow-up did not differ between the groups of former USSR immigrants 20.9y (95% CI 19.5–22.3), and Israeli-born 21.3y (95%CI 20.5–22.1, *p* = 0.7) but was shorter among the immigrants from other countries 18.9y (95%CI 17.0–20.8) as compared with the Israeli- born groups (p(Chi square = 5.6) = 0.03) (Fig. 3). When stratified by the 3 periods, a significant difference existed in the middle period (p(Chi square = 5.2) = 0.02) only. The age of death was significantly younger among the former USSR group 44.2 ± 9.2 years (Russian Federation 44.0 ± 9.6, Ukraine 45.6 ± 8.3, others 40.7 ± 9.4) than the other immigrants 56.8 ± 10.9 and Israeli-born 54.3 ± 10.2 (p(F = 14.0) < 0.001). Age of admission differed significantly between groups (Table 1), but duration from admission until death was comparable (9.2 ± 6.1, 10.6 ± 7.2 and 10.3 ± 7.1 respectively, *p* = 0.4). The cause of death (Appendix) among the Israeli-born group was primarily cancer (20.9%) followed by overdose intoxication (9.5%), while among the USSR immigrants, overdose was the most prevalent cause (13.2%) followed by HIV (9.2%).

## Discussion

In this study, we compered the former USSR immigrants’ characteristics and outcomes in MMT with other immigrants and Israeli-born patients. Overall, the former USSR immigrant patients have a better outcome in MMT, as reflected by the longer cumulative retention since their first admission to MMT, than other immigrants and Israeli-born patients. Retention, as a good predictor for success in MMT, is influenced by various factors that can be social, program, and individual. Interestingly, in the current study, the former USSR immigrants were characterized with some variables known to associate as predictors for longer retention, but others are known as associated with poor retention. Specifically, the former USSR immigrants, in multivariate analyses as compared to the Israeli-born group, characterized as more educated (+ 12 years of education), were admitted to treatment at a younger age < 30 y following shorter duration (< 20 years) of opioid usage, a higher proportion among them were of non-Jewish faith. Most of them (both Jews and non-Jews) were secular. This may reflect the fact that in the USSR religion was banned, and atheism was promoted at the state level during a very long (nearly 70 years) period. More of the former USSR immigrants had no children, had a history of drug injection, and indeed more of them had positive antibodies for hepatitis C and HIV. Also, more of them had a history of alcohol use. In addition, more of them had no DSM-5 Axis I&II psychiatric diagnosis, and more of them had only DSM-5 Axis I diagnosis only. Although we don’t have information on whether it was prior to- or since- immigrating to Israel, the history of criminal involvement was also lower among the former USSR immigrants. It should be noticed that the criminal activities since entering MMT were comparable and minimal for all patient groups (data not shown).

Of the sociodemographic factors, a review covering 19 studies among Chinese MMT clinics found older age, having, or regaining a job, being married, being highly educated, female gender, and having religious belief as predictors for longer retention [11]. Having a job was found in a study from Canada, to be the strongest predictor, [12]. Another study from Canada found that recent homelessness and incarceration, not accessing social income assistance, daily prescription opioid use, and daily heroin injection drug use predict poor retention [23].

A review study from Vietnam found individual patients’ factors such as sociodemographic characteristics, substance use before and during treatment, economic status, awareness of HIV and MMT, co-occurring disorders and comorbidities, and the length of treatment time to be significant. While older age was associated with longer retention, interestingly, higher education level in this study group was related to poor retention, explained as they might have more employment and social network opportunities, which may conflict with the medication schedule, as well as worse retention of married persons [24].

Higher education (> 8 years of schooling) is considered a factor positively affecting retention rates [3]. The former USSR immigrants’ group in our study is more educated (12y +) than the other groups, supporting previous studies that examine the association between education and retention. History of fewer criminal events also characterized the former USSR immigrants, another factor associated with more years of education. Most MMT studies that examined age found that older age at admission (> 30) is associated with increased retention [5, 11, 22], a finding that reflects patients’ long-time experience of abstinence failures and continued suffering, which leads to a more mature state. Still, the former USSR immigrants’ group is characterized by younger age at admission to treatment (35.7 ± 9.2). Also, contrary to studies supporting the hypothesis that fewer years of drug use before admission to MMT predicted shorter retention [11], the former USSR immigrants’ group findings in our study suggest the opposite. In the current study, the longer retention group following a shorter duration of opioid usage (20y + was among 27.1% only compared with about 50% in the two other groups) predicted a better outcome.

The overall positive outcome of this cohort of immigrants from the former USSR (during the first years of the 1990s) admitted to the MMT program not too long after having arrived in Israel may be attributed to a combination of their characteristics: a group of young, educated persons, with a relatively short duration of opioid usage (in many cases opium), with only a few or no criminal history, who arrived to their new country where they had, for the first time, the access to an MMT program (not available in their country of origin, hence with no previous stigma regarding methadone). This combination of characteristics luckily saved them the long-time frustrating experience of repeated detox trials and abstinence failures.

MMT cohort studies that examined substance use’s effect on treatment outcomes found that polydrug misuse at admission is a risk factor for reduced retention. Those studies scrutinized different kinds of drugs such as cannabis, amphetamines, benzodiazepines, cocaine, and heroin/opiates, and found that all of them were associated with poor MMT outcomes [22, 25–28]. Amount of use, frequency of use, and way of using also affected retention. Higher drug use frequency predicted shorter MMT retention. Besides, less drug use per day predicted longer retention [11]. Methadone dose itself influences treatment outcomes. The methadone dose prescribed should be able to prevent withdrawal, block craving and stabilize the patient, and it has been noted in previous studies that a high dose (> 100 mg/d) of methadone has positive effects on longer cumulative retention [25].

Studies dealing with drug injections did not find an association between injection and retention; Ledgerwood et al. [29] studied whether injection use is associated with MMT outcomes and found injection to be associated with cocaine use but not with opioid and length of treatment retention. Mullen et al. [30] studied factors that can indicate the length of retention in MMT and found no association between retention and injection status or sharing injecting equipment. In the current study, the former USSR immigrants who mostly had a history of drug injection (86.2%) had longer retention. Conversely, studies that examined the association between alcohol abuse and retention rates found that patients with pre-treatment alcohol problems have a higher chance of dropping out [28, 31]. Surprisingly, in our study, alcohol pre-treatment use is greater (79%) in the longer retention group. Again, an explanation of the former USSR immigrants’ success is as they had no MMT in their native country, they were more engaged when offered the opportunity to be admitted to an MMT program in Israel, independent of their injection or alcohol history. However, it should be kept in mind that Ukraine did establish a maintenance treatment program in 2004 [32] which was later banned in the occupied territories by the invading army of the Russian Federation (Crimea February–March/2014, Donbas and other Eastern parts of Ukraine February/2022) [33].Thus, in our cohort only 3 patients from Ukraine immigrated between 2004–2013, and 1 between 2014–2022.

Supporting our findings of superior outcome for the former USSR group is a report that studied factors that characterized unplanned early discharges from a Dual Diagnosis Inpatient Detoxification Unit in Israel [34], USSR immigrants had increased rates of completing the program as scheduled.

Looking in depth among the former USSR immigrants, most of them, in our cohort, initiated drug usage before emigration. In a study among former USSR immigrants in Israel, half of them had SUD before emigration (many of them to opium, which some had started using during their military service in Afghanistan [35], while others lived in the Asian Republics of USSR, near the Afghan or Iranian borders). The median age of substance use was 32.0 years [15]

A study from France on migration from Eastern Europe, particularly from ex-Soviet Union countries, found that the great vulnerability of the participating population was often attributable to the loss of social status after migrating to France. Participants had better access to harm-reduction tools and reduced their risk of exposure to HIV and HCV infections linked to needle sharing [36].

While survival since admission to MMT was comparable between our study groups even though former USSR patients were admitted to MMT at a younger age, the age of death of patients from the former USSR was significantly younger than that of the Israeli- and immigrants from other countries. These findings support several reports of a shorter life expectancy of the former Soviet Union populations (now divided among many various countries, but with similar or comparable life expectancy reported by both the World Health Organization and the World Bank Group [37, 38], than that of Israeli-born, or western countries-born persons. As would be expected from the differences in age of death, while the cause of death among the Israeli-born group was primarily cancer, the former USSR immigrants primarily died due to overdose and HIV (to emphasize that all our patients with HIV are being treated and followed up in the Clinical Immunology Unit in the hospital and the medications are available to all through the National Health Services, but individual adherence with the recommended treatment may not always be optimal, as is possibly the case of those who died of AIDS). However, death numbers are too small to generalize, and additional follow-up is needed.

Concerning dual diagnosis, fewer of the former USSR group had a dual diagnosis; however, the difference was attributed to females. The males in this group did not significantly differ from the Israel-born group. Interestingly, many male former USSR immigrants were Veterans of the Soviet Afghan war (1979–1989). Had they returned to a Western World country after the war, many of them would have been diagnosed with post-traumatic stress disorder (PTSD) [39]. Nevertheless, this diagnosis did not exist in the former USSR, and the survivors were not even aware of the possibility of being affected by that (for them) “a non-existing mental condition”, hence did not seek treatment even after having immigrated. While the strength of the study is the longitudinal cohort, its weakness is the small number of immigrants among the entire cohort. Another limitation is that while we had specific information on several personal factors before and since admission to MMT, we had no information regarding these factors during the periods pre- and post-immigration (before admission to the program).

### Limitation

One of the limitations of our study is that we included only variables that were available to most or all the patients’ cohort, and did not check specifically about mental and physical well-being and work status during treatment. However, the findings are meaningful, as retention rate is a core factor for the success of the maintenance treatment and is known to reflect patients' success.

We must limit our conclusion with respect to death age and causes as the number is too small to generalize, and additional follow-up is needed.

Further in-depth socio-cultural qualitative studies that would conclude on psychodynamic reasons for patients' behavior, would enable better understanding and maybe operative treatment results.

## Conclusion

Former USSR immigrants arrived in Israel mostly during the first years of the 1990s following the dissolution of the Soviet Union (more than one million people, many of them from the Russian Federation or Ukraine, others from the Caucasus countries or the Asian Republics), and despite several characteristics that are known to associate with poor outcome, had the best adherence to treatment as reflected by their longer cumulative retention in their first admission, with lower rates of readmission. Overall, since the first admission and until the latest one (including duration out of treatment), retention in treatment was comparable to the Israeli-born group. Intensive medical and psychiatric intervention to prevent young age deaths is recommended.

## Figures and Tables

**Fig. 4:**
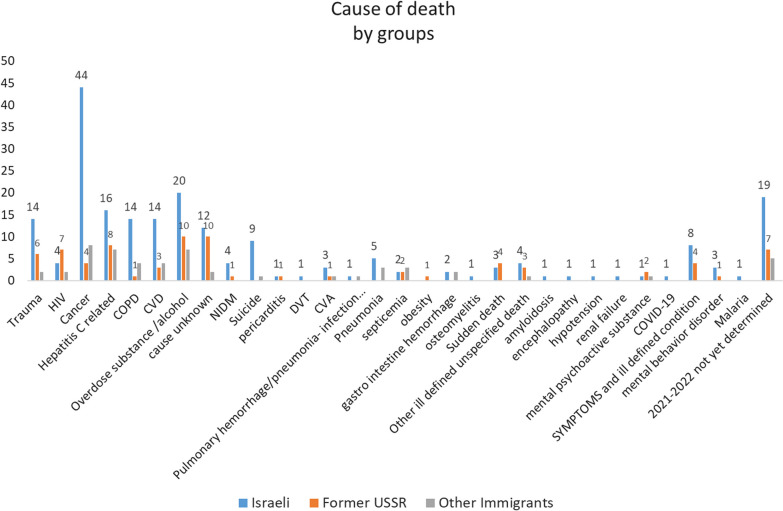
Cause of death number by former USSR, other immigrants, and Israeli-born subgroups

## Data Availability

Data cannot be shared openly, but available from the authors upon reasonable request.

## Appendix

See Fig. 4.
